# Database of historic ports and coastal sailing routes in England and Wales

**DOI:** 10.1016/j.dib.2019.104188

**Published:** 2019-07-02

**Authors:** Eduard J. Alvarez-Palau, Oliver Dunn

**Affiliations:** a Universitat Oberta de Catalunya, Spain; b University of Cambridge, UK

**Keywords:** Ports, coastal routes, sailing ships, transport infrastructure, historical geography

## Abstract

This data paper presents a reconstruction of historical ports and coastal routes in England and Wales during the age of the sailing ship, ending at the beginning of the twentieth century. The dataset was created by an amalgamation of twenty different sources, including geographical data, primary sources and secondary literature. Ports found in historical documents were listed by year of appearance and georeferenced. Ports that appear in multiple sources were listed only once. Coastal routes between ports were drawn based on navigation charts and bathymetry data, distinguishing five categories with different characteristics. Visibility from the coast was deduced from elevation rasters and lighthouse locations. Subsequently both ports and coastal routes were checked using topological rules to ensure the connectivity of the network. The data is provided in shapefile format with all the attributes and can be analysed using Geographical Information Systems (GIS) for different types of geographical and historical studies.

**Table:** Specifications table

Subject Area	Human Geography
More specific subject area	Historical Geography, Transport networks
Type of data	Tables, graphs and figures
How data was acquired	We relied on primary and secondary sources of ports and coastal routes. For primary sources we digitised them ourselves from the corresponding archive.
Data format	Raw, filtered and analysed data
Experimental factors	The data was manually compiled, transcribed, standardised, and georeferenced. It covers a period of over three hundred years, between the mid-sixteenth and early-twentieth centuries.
Experimental features	We built our historical database using Geographic Information Systems (GIS), including different attributes for each feature.
Data source location	England and Wales
Data accessibility	E.J. Alvarez-Palau, O. Dunn, D. Bogart, M. Satchell, L. Shaw-Taylor, Historical ports and coastal sailing routes in England and Wales 1540–1914. Data Collection]. Colchester, Essex: UK Data Service – Reshare, 2019, https://doi.org/10.5255/UKDA-SN-853711
Related research article	D. Bogart, O. Dunn, E.J. Alvarez-Palau, L. Shaw-Taylor, Speedier delivery: coastal shipping times and speeds during the age of sail, unpublished results. O. Dunn, A sea of troubles? Coastal shipping speeds in seventeenth-century England and Wales, unpublished results Sacks, D.H. & Lynch, M. (2016): Ports 1540–1700. In Clark, P. The Cambridge Urban History of Britain. Cambridge University Press [1]

**Table:** 

**Value of the data** • The data aims to contribute to a better understanding of the transformation of transport infrastructure during the industrial revolution, especially where this concerns changes in coastal shipping services and port facilities. Research can be undertaken into regional economic change in the long run, given the broad geographical and temporal coverage of the data. • Spatial and network analysis can be performed using Geographical Information Systems (GIS). Sailing route distances between ports can be calculated. The combination of separate sources of data for inter-port sailing times allow for estimations of shipping speeds. Changes in recorded speeds can be estimated over time and compared with speeds elsewhere. • The effect of new technologies and investment can be assessed. Shipping services can also be examined under different circumstances, such as at night or in daytime hours. • Ports can be assigned qualitative attributes (e.g. for a wet dock) and available infrastructure (e.g. for cranes) for different historical periods. Additional attributes, such as traffic, flow, or meteorological data, can also be linked with the database of coastal routes to allow for new analyses. • External data for different transport modes can allow for the integration of ‘multimodal’ transport networks, covering different time periods.

## 1 Data

The data described in this paper consist of two main GIS shapefiles:

1) A dataset of historic ports
2) A dataset of historic coastal sailing routes

**Fig. 1:**
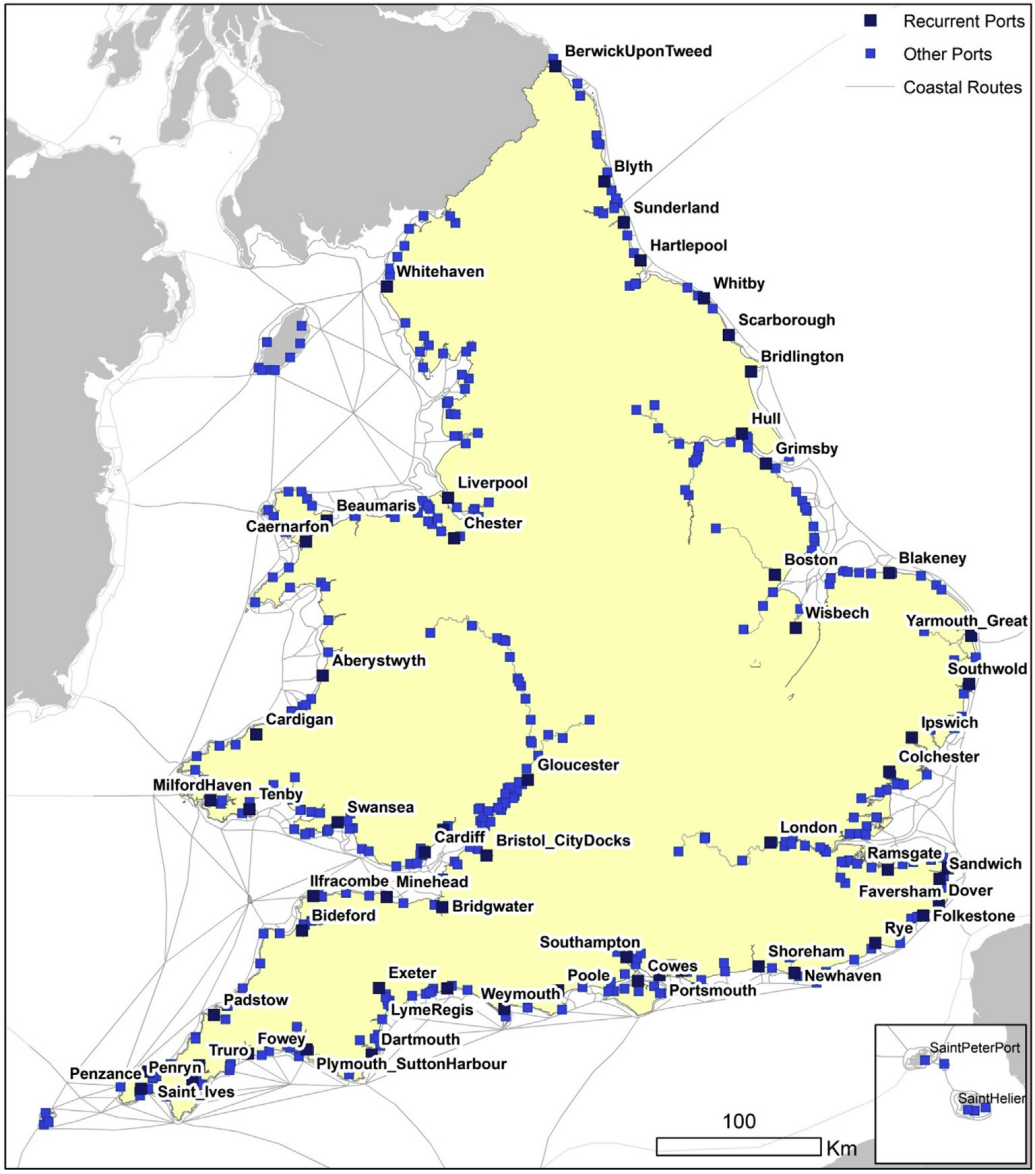
Ports with one or more mentions within the sources used. Those places marked as dark blue (recurrent ports) are mentioned in at least nine out of eleven sources.^5^ Own work.

Both shapefiles were created using a combination of GIS techniques and historical research that drew on several primary and secondary sources (Table 1). The aim was to recreate, as accurately as possible, historic sailing routes (Fig. 5) and port infrastructure (Fig. 1) of the coastal shipping network of England and Wales.^1^ The data cover a period of over three hundred years between the mid-sixteenth and early-twentieth centuries.

**Table 1:** Sources with lists of historical ports in England and Wales.

Year	Source	Number of ports
1565–1700	Sacks, D.H. and Lynch, M. (2016): Ports 1540–1700. In Clark, P. The Cambridge Urban History of Britain. Cambridge University Press [1]	110
1575–1765	Wanklyn, M.D.G., Wakelin, P., Hussey, D. and Milne, G. (1996). Gloucester Port Books, 1575–1765. [Data collection]. UK Data Service. SN: 3218, http://doi.org/10.5255/UKDA-SN-3218-1[15]	131
1603–1840	Bogart, D. and Richardson, G. (2011): Property rights and parliament in industrializing Britain. The Journal of Law and Economics 54.2: 241–274 [9]	92
1651–1683	Sample of 6,933 entries from Coastal Port Books (E122 & E190)	180
1680	Hargrave, F. (1787): A Collection of tracts relative to the law of England from manuscripts, Vol. 1. Dublin. 650p [10]	131
1780–1914	Langton, J. & Morris, R.J. (2002): Atlas of Industrialising Britain, 1780–1914. Taylor & Francis [8]	93
1826	Steel, D. (1826): Ship-master assistant and owner's manual. London [11]	183
1830–1845	Sample of 4,146 entries from Board of Trade Crew Lists (BT98)	220
1842	Daniel, J. (1842): The Shipowner's and Shipmaster's directory to the port charges, all the depth of water in Great Britain and Ireland. Aberdeen. 269p [12]	247
1903	Hopwood, F. (1903): Harbour authorities. Return from the authorities of the harbours, &co. of the United Kingdom. London: Eyre and Spottiswoode. 286p [13]	164
1911	British Parliamentary Papers (1911). Coal Shipments. Tables giving details as to shipments of coal abroad, coastwise, and as bunkers, from each port of the UK. Cd 5647. London: His Majesty's Stationery Office [14]	77

The data can be used to better understand long-term effects of transport infrastructure on social and economic history – specifically from maritime trade [2], [3], [4], [5]. Potential applications include shipping transport modelling [6], [7] and research into the development of infrastructure, such as piers, lighthouses and docks, among other developments.^2^

### 1.1 Data on ports

Drawing on eleven different sources, we created a list of ports and other smaller places where coasting vessels regularly landed to load and discharge goods. The existing literature provides convenient lists of the most important ports. However, coasting vessels called at a much larger range of landing locations than these suggest – including beaches, natural bays, etc. To locate and record more places we drew on new sources that provided us with an array of landing locations at different benchmark dates.^3^

Table 1 indicates the date range covered by each source, the reference to the publication, and the number of ports we derived from each and included in our database. We worked with secondary sources when looking for port listings, such as Ports 1540–1700 [1], *The Atlas of Industrialising Britain* [8], and 'Property Rights and Parliament in Industrializing Britain' [9]. We also used primary sources, including printed publications [10], [11], [12], [13], government records [14], the Gloucester port books database [15], and our own data harvested from other port books and from 'crew lists' found in the Board of Trade.^4^

Digitising port information from secondary sources was relatively easy. What was more difficult was gathering port data from the port book and crew list coastal shipping data. Both sources give the movements of coasting ships and, as a result, also record myriad landing locations and ports that often do not appear in the secondary sources. These ports were included in the port data presented here.

The British government's board of trade collected thousands of crew lists from ships to keep track of mariners, and between 1830 and 1844, their lists also recorded ship movements. The lists survive in good numbers, so we were able to sample randomly from this source.

From 1565 to 1790, customs officials at regional ‘head ports’ documented ship movements in their domicile port and in smaller local creeks and havens using the standardised port book volumes. Over twenty thousand port books created by this early modern fiscal system survive. Yet, it is difficult – perhaps impossible – to undertake national level random sampling due to patchy survival rates and the current removal of most books to the conservation department at the National Archives, Kew. Therefore, we drew on a non-random sample of port books from the seventeenth century. We collected all useable data from a port book set originally created during the Cromwellian or Interregnum period, 1649–1660 (E122). This set includes numerous ports located on the West, South and East coasts of England and Wales. It comes from the records of 13 customs ports. However, the thousands of coastal voyages these volumes recorded include detail for a much larger set of places because officials also recorded the varied and numerous destinations and ports of origin of coasters. Later, to strengthen our sample of eastern and western ports, we added sets from the port books of King's Lynn and Barnstable to 1683, this time taken from port books still available in the main E190 port book series.

Because of geographical sampling limitations, the port book data we found cover coastal trades that varied in type and by region. Our sample cuts across several different trades with different characteristics. Riverine trade of small vessels of around 15 tons, for instance, are common in Gloucester's port book data [15], whereas our own set includes larger ships that traded in the North Sea. Forty-six percent of the first sample comes from Essex and Lincolnshire ports and the ports connected to them by their coastal trade routes. Most of their trade was in fact oriented to London. This particular sample recorded ships that carried cargoes including coal and grain and to various port locations, including the smaller ports of Spalding and Maldon and larger ports like London and Newcastle.^6^

In the nineteenth century, the number of reported ports of all kinds increased compared with the sixteenth century due to better information in the sources, but also because of the overall expansion of the network of coastal ports. According to earlier sources, 'ports' included harbours, small creeks or just beaches. These were overseen by larger ports with customs houses. Fig. 1 shows the geographical distribution of all 479 ports that appear at least once in our sources.^7^

### 1.2 Data on coastal routes

In the sailing era, natural conditions constrained operations, especially storms, tides and waves, but also low light, and clearly these all had adverse effects. In terms of navigation, instruments used at the time allowed travel only under certain circumstances, and good visibility was necessary for safe passage. Knowledge of bathymetry was key to avoiding damage by grounding on sandbanks or rocks. Navigational charts reported the depth of water at certain locations, but for these to be of any use it was crucial to know the exact position of the ship. Mariners used landmarks to track their position, often using triangulation, and it was thus normal to sail in sight of the coast. During the night, or in poor visibility, navigation became difficult. Beacons, lighthouses and light-vessels etc. served as an alternative to landmarks where available, but their presence on the coast was very limited at the beginning of our period of study.

We used an amalgamation of different sources to identify coastal routes mariners most likely followed. Specifically, we relied on historical coastal charts, bathymetric depth rasters, topographic elevation rasters, and parliamentary reports to create our database.

The main primary sources used to determine coastal routes were navigation charts included in Captain Collins' publication, *Great Britain's Coasting* *Pilot*, first published in 1693 [16]. These documents were digitised and geolocated to gain a workable understanding of the contemporary navigation techniques of each period. Charts always contain landmarks and bathymetry information so the mariners who used them could determine their position and avoid danger. Collins also gave specific directions for some routes with their distance in miles given, and this information revealed the routes the author directed ships to take when sailing round the coast.

Bathymetry data was used to distinguish those areas with sandbanks or submerged rocks. Although we understand the position of sands changed over time, we assume there was historical stability in other parts of the coast that were less affected by tides and oceanic currents. We relied on the EMODnet Bathymetry data for the Atlantic Ocean, published by the European Marine Observation and Data Network in 2016 [17]. Specifically, we obtained a Digital Terrain Model (DTM) raster with bathymetric depth data with an approximate resolution of 200-m cell.^8^

Topographical data were gathered from the NASA Shuttle Radar Topography Mission (SRTM). Our raster, however, was a processed version offered by the International Centre for Tropical Agriculture (CIAT); in particular, we worked with its version 4.1 [18]. In this case, the different rasters were provided in TIFF format with a resolution of 90-m cell.

Finally, we also used five sources to obtain the location and visibility range of lighthouses, beacons^9^ and light-vessels. Collins’ *Coasting* *Pilot* [16] was used for the first period because it shows their location and detailed visibility ranges for night-time navigation. It was reviewed and complemented secondary sources [19], [20]. For the second period we used The *Light-Houses of the British Islands* in two editions, one published in 1832 and the other in 1851 [21], [22].

## 2 Experimental design, materials and methods

Using all these sources, we created two databases covering all of England and Wales. The database of ports was designed as a shapefile with georeferenced points and temporal data. When we compiled the port data, we maintained full linkage between ports and all sources so users can view ports by specific source dating from chosen period. This allows the ports database to be used in time-dynamic analyses because ports that were in operation at given periods of time between the mid-sixteenth to the early-twentieth centuries can be isolated from those that were not. The shapefile of coastal routes interconnects the elements of the previous database, distinguishing each segment by category. We also introduced time dynamic data for lighthouses to show building date and changes to their characteristics like light visibility range. This gave us a more precise account of a key change in navigational visibility over this period.

In geographical terms, both shapefiles were created using the coordinates system of the British National Grid projected into Transverse Mercator with datum ODGB 1936 of the Ordnance Survey National Grid.

### 2.1 Database of ports

The shapefile of ports includes precise information to locate each element geographically and temporally. Table 2 shows the set of fields included in the resulting shapefile.

**Table 2:** Attribute table of the database of ports.

Field	Data type	Description
FID	Object ID	Unique ID for each row in the table
Shape	Point	Point for location of the port
Name	String	Unique name of each port
Sac1565	Numeric	Indicator = 1 if port is identified by Sacks and Lynch (2016) for the period 1565–1700
Wnk1575	Numeric	Indicator = 1 if ports is identified by Wanklyn et al. (1996) for the period 1575–1765
Bog1603	Numeric	Indicator = 1 if ports is identified by Bogart and Richardson (2011) for the period 1603–1840
Cpb1651	Numeric	Indicator = 1 if port is identified in our sample of Coastal Port Books for 1680
Har1680	Numeric	Indicator = 1 if port is identified by Hargreave (1787) for 1680
Lan1780	Numeric	Indicator = 1 if port is identified by Langton and Morris (2002) for 1780–1914
Ste1826	Numeric	Indicator = 1 if port is identified by Steel (1826)
Bot1830	Numeric	Indicator = 1 if port is identified in our sample of Board of Trade for 1830
Dan1842	Numeric	Indicator = 1 if port is identified by Daniel (1842) for 1836
Hop1903	Numeric	Indicator = 1 if port is identified by Hopwood (1903)
Bpp1911	Numeric	Indicator = 1 if port is identified in British Parliamentary Papers (1911)
Bog_Acts	Numeric	Number of harbour acts taken from Bogart and Richardson (2011)
Bog_First	Numeric	Year of first harbour act taken from Bogart and Richardson (2011)
Point_X	Numeric	X coordinate of the port
Point_Y	Numeric	Y coordinate of the port

Fields *FID* and *Shape* were automatically generated by the software. The first indicates the order in which each element was recorded. The second indicates the type of element, in this case, points.

Field *Name* was created as a unique identifier. It allows the linking of each element, often named differently across different sources, with the same register of all the other sources contained in our database.

The following variables contain information on the presence of each element in every source. In each column we used a binary variable, identifying those elements that were listed in that source (value = 1) and those that were not (value = 0).

Finally, fields *Point_X* and *Point_Y* include geographical coordinates of each element based on the British National Grid. Please note that the geolocation of each point was done manually and with maximum precision given the available information. In many cases, we had to rely on current maps, such as ESRI's World Imagery system or Google Earth to find and locate named places. This should not pose a problem since we do not expect any significant variation in their location over the period of study.

#### 2.1.1 Descriptive statistics

**Fig. 2:**
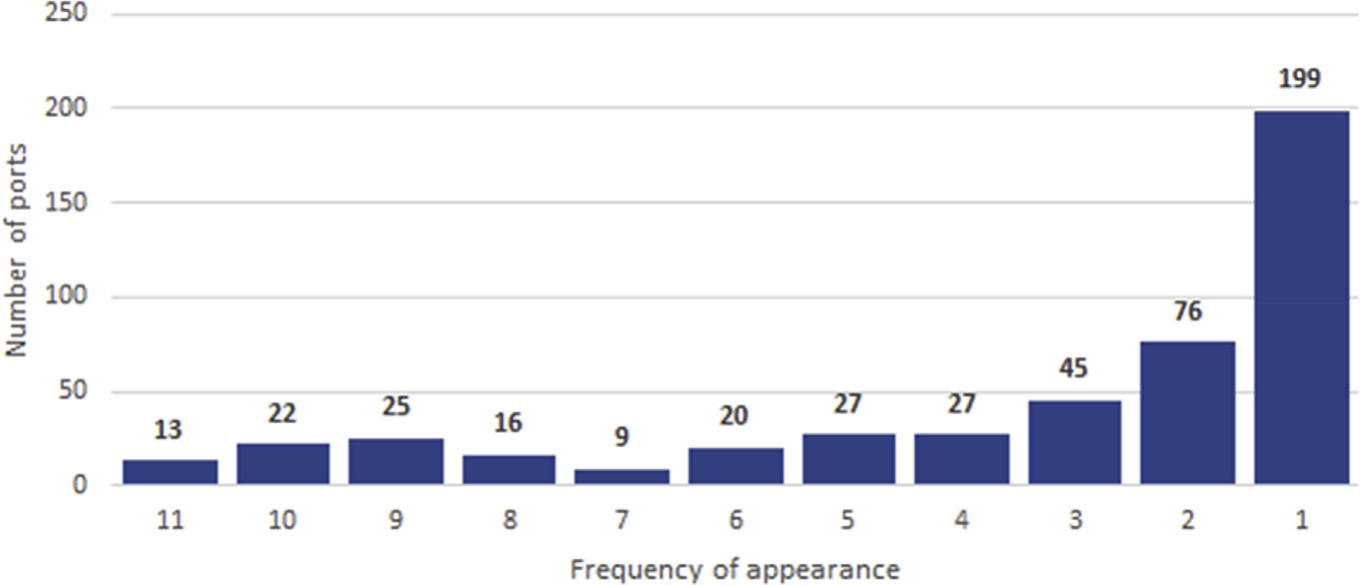
Number of appearances of each port across different sources. Own work.

In summary, our ports database includes 479 different places. Only 13 ports came up in all 11 sources.^10^ These appear to be consistently important places for shipping over the period of study. Others appear in the data in different benchmark years, which indicates real changes in use, but also distinct recording practices at different times and between the sources. Fig. 2 shows the aggregate distribution of the number of appearances of each port in all sources.

Note the relative stability of the number of appearances, except for the long tail of ports with only one, two, or three observations. These include smaller ports that go unreported in published sources, but that were nonetheless visited by real coasting ships.

#### 2.1.2 Temporal coverage of the sources

**Fig. 3:**
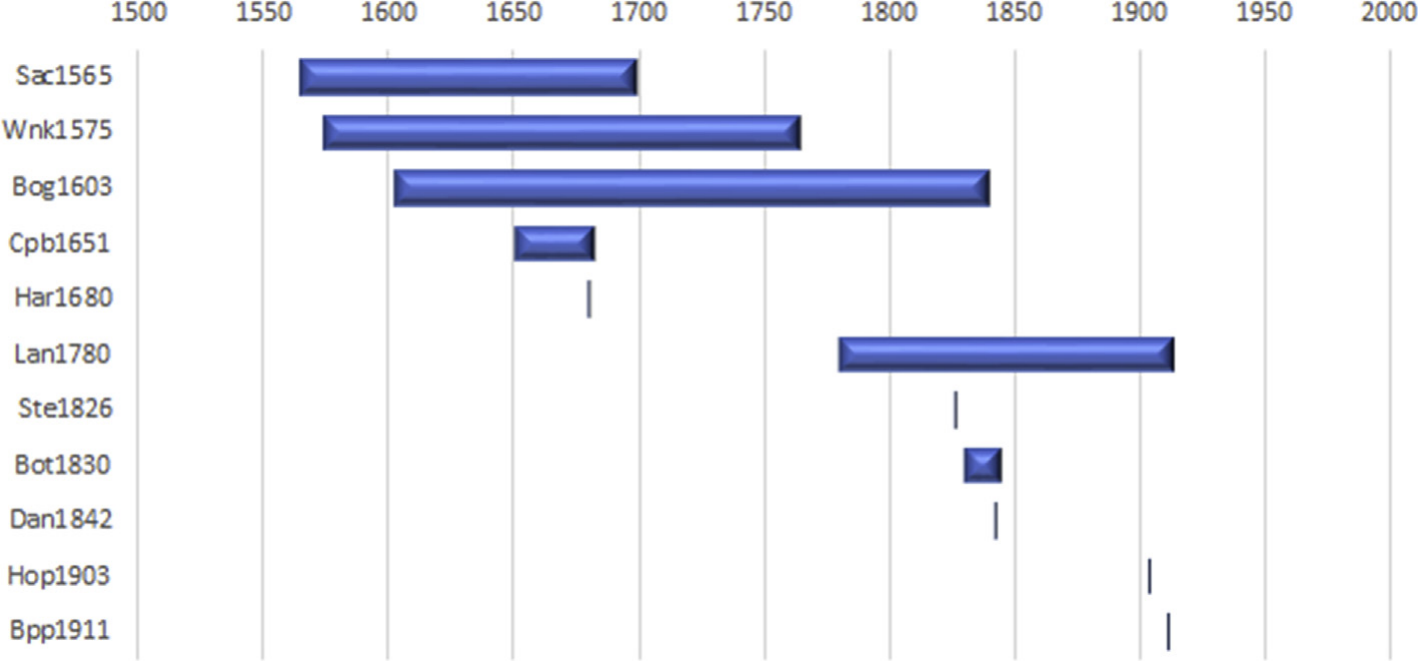
Temporal coverage of each source considered in our study. See Table 2 to identify each acronym to its source. Own work.

In our work, we use data from many different sources, and each source has its own temporal coverage. Some sources, for instance, list ports in operation at specific dates. In others, data was compiled at some point from a specific archive over a longer period. In these cases, the presence of a specific port on the list does not imply that port was in operation for the whole period, but just for the time it was recorded. Fig. 3 shows the temporal coverage of each source.

Observing the chart, one can see that the sources pertain to different periods. Aggregate lists of ports dating from before the eighteenth century do not exist to our knowledge. For later periods, comprehensive lists of ports for specific periods are available. In these cases, the sources provide more precision; however, diversity and coverage are limited by a focus on larger ports often with specific infrastructure or facilities.

#### 2.1.3 Improvement of harbour infrastructure

Wet docks did not exist in England and Wales in 1660, and only a handful of harbours had been built by this time. Many ports, however, were greatly improved by the beginning of the twentieth century. It is estimated there were 391 acres of wet dock space and 50 harbours in 1830 [23]. The authority to improve ports came largely from Parliament. Acts of parliament often created a public trust or private corporation with powers to charge tolls and build or repair harbours, piers, or docks. Often these powers were amended by subsequent acts, and some port authorities were affected by numerous acts.

**Fig. 4:**
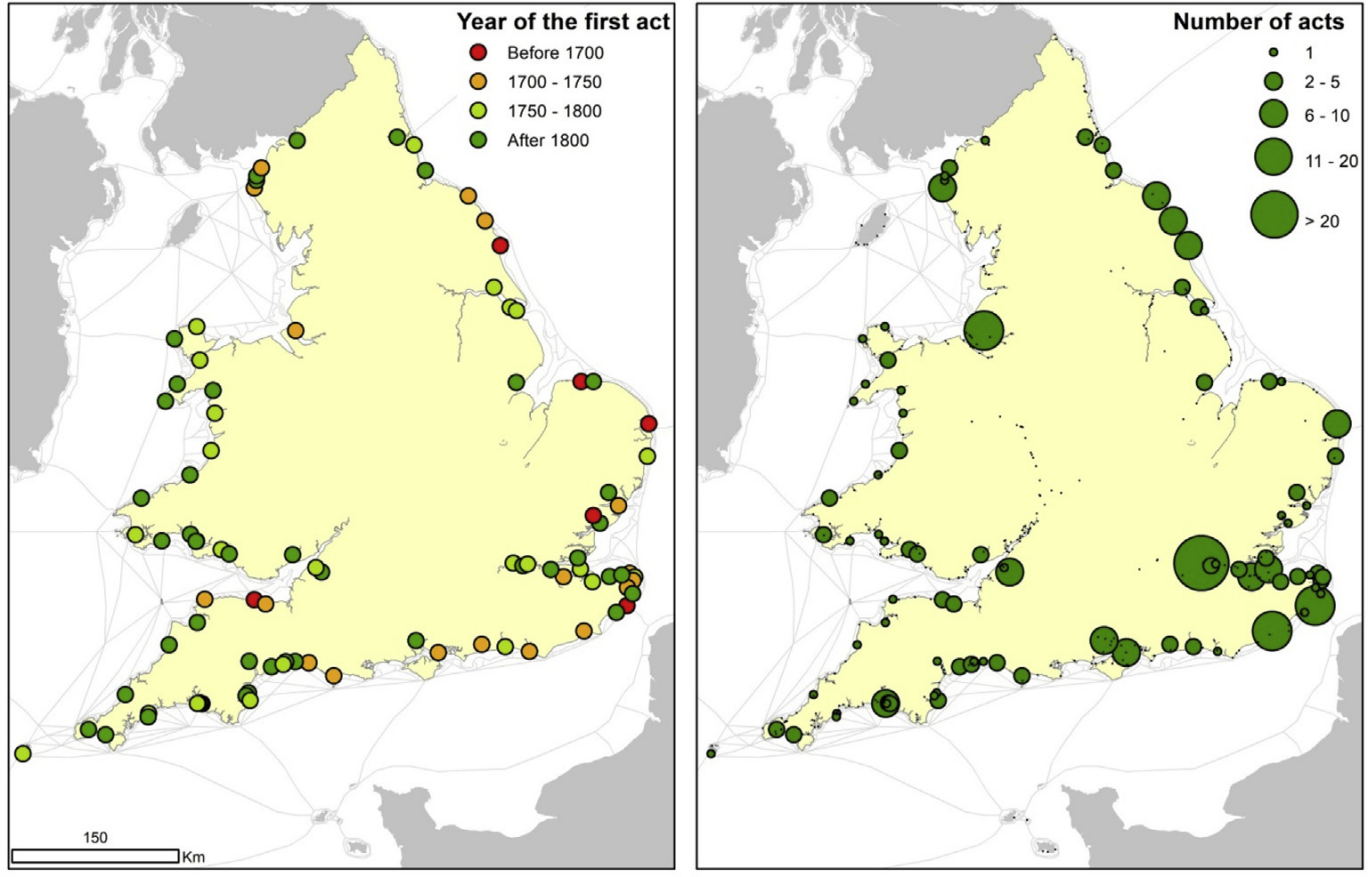
Ports with harbour acts granted by parliament. On the left panel, ports are classified according to the year of their first act. On the right panel, the size of the dots is plotted according to the number of acts involving each port. Own work.

Bogart and Richardson [9] collected a list of all acts granted to improve port infrastructure between 1660 and 1840. The list contains the title of the act and the year it became law. Importantly, the title includes the name of the port, which we used to link all port acts to our database of ports. The dataset also provides a count of all acts affecting each port in different periods between 1660 and 1840. Both attributes are plotted in Fig. 4. Note that there are many zeros, which indicate that a new trust or corporation was not yet created for a port between 1660 and 1840. A zero probably indicates little improvement to the port, although the details should be checked in individual cases.

### 2.2 Database of coastal routes and connections

The database of coastal routes was designed to interconnect the database of historical ports. We opted not to draw direct connections between individual ports, but rather to introduce interconnected segments with different characteristics through which all connections could be made. The structure of the attribute table is summarised in Table 3 below.

**Table 3:** Attribute table of the database of coastal routes.

Field	Data type	Description
FID	Object ID	Unique ID for each row in the table
Shape	Point	Point for location of the port
Type	String	Typological classification given to the polyline
ViNig1690	Numeric	Indicator = 1 if the polyline is within the visibility range of any lighthouse in 1690
ViNig1830	Numeric	Indicator = 1 if the polyline is within the visibility range of any lighthouse in 1830
ViDay	Numeric	Indicator = 1 if the polyline is within the visibility range of the coastal skyline
Length	Numeric	Length of the polyline in metres

The software automatically generated fields *FID* and *Shape*. The first records the order in which each element was added. The second indicates the type of element, in this case, polylines.

**Fig. 5:**
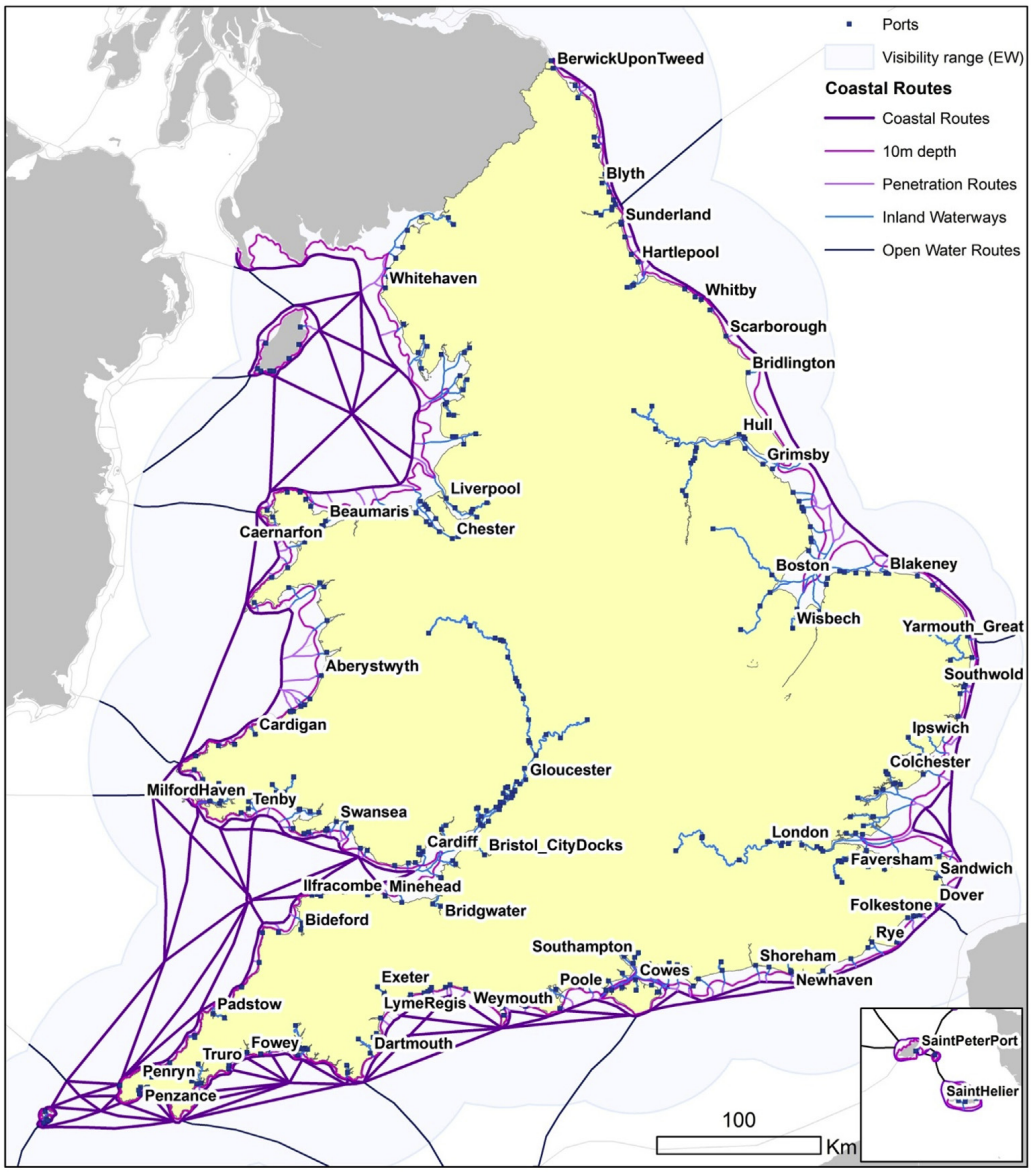
Coastal routes classified by category. Own work.

Field *Type* indicates the category of each polyline. It allows the classification of the routes by their nature. We opted for five different levels of classification, as seen in Fig. 5:

Level 1: Coastal routes based on maritime navigational charts.

The charts detailed coastal landmarks which mariners could use to determine their geographical position. The inclusion of landmarks aided the description of coastal routes, such as that between London and Newcastle. Directions should not be understood as being precise, but more like broad corridors ships could use to navigate safely between geographical points. Nautical charts also estimated distances between landmarks, which enabled navigators to plan their route and to calculate travel times using the point-to-point distances provided on pages of text interspersed between the bound maps.

We were able to recreate the primary coastal routes described in this source in ArcGIS by geolocating landmarks and plotting routes between them according to the various given orientations, and by comparing them with the distances quoted in the source. Furthermore, we ensured all the connections were within the visibility range from the coast in good weather and in daylight.

Level 2: 10m depth bathymetry isolines.

Assuming ships could become grounded when the depth of the water was less than the draught of the ship itself, nautical charts tended to report accurate bathymetry data. It allowed ship masters to circumvent those areas with greater ruggedness and to avoid danger. In this sense, the distance from the coast became an issue for navigation. Visibility of the coast, especially in poor weather, required proximity to the shore. However, the depth of the seabed near the coast was a clear constraint. Mariners had to remain close enough to shore to see landmarks and sufficiently far out to avoid grounding.

To incorporate this issue into our GIS model, we drew a continuous line around the coast of England and Wales at a 10m constant depth. To do so, we relied on the EMODnet bathymetry raster [17] described above.

This line represents the closest a ship could navigate to the coast in order to avoid sandbanks and underwater rocks. Ships may have had draughts of less than 10m, but given the limited precision of the bathymetry raster employed, we believe this value is a safe threshold estimation.

Level 3: Penetration routes to port.

As described in the previous category, in order to avoid running aground, navigation in shallow waters was not recommended, but ships had to access and depart from ports located on the coast. In many cases, the average depth of the seabed around ports was much lower than 10m and the route to enter them was riddled with obstacles.

To solve this problem, we drew penetration lines connecting ports with those routes rounding the coastline. These lines were meant to maximise the depth of the seabed from the port to the 10m isolines and the coastal routes, as described above, for which we used [17].

Level 4: Inland waterways.

Some of the ports recorded were not located on the coast, but on inland waterways. We cannot assume, based on our data, that these ports were accessible from the coast without transferring goods to smaller riverboats, yet we know there were regular coastal trading connections with river ports. At this stage, this allows for a basic overview of the extensive interconnection of inland waterways and coastal routes for transportation.

For this exercise, we plotted those rivers, lakes, and sea-lanes required to guarantee connection to all our ports and landing locations. Note that the set of these lines was not conceived to include all inland waterways, but just those needed to ensure the interconnection of our coastal network as it is detailed by the sources used [24]. The Gloucester Port Books Data [15], for example, provided hundreds of ‘coastal’ ports dating between 1575 and 1765, some of which were located upriver and far from the sea. Despite this, the source shows they were integrated with the broader coastal trade, and thus we connected them with the rest of the coastal network model.

Level 5: Open water routes.

Some routes described in our sources exceeded the ranges of visibility from the English and Welsh coasts (i.e. connections to Ireland, Scotland, and the Channel Islands). For those cases, and because lines categorised in Level 1 were restricted to be within the daylight visibility range from the coast, we drew additional lines representing these connections.

The following three fields give information on the visibility of the coast from each route. The first field corresponds to daylight visibility, while the later ones refer to night visibility in the late-seventeenth and mid-nineteenth century.^11^ All three fields were defined as binary variables with a value equal to 1 when the route was within the visibility range, and a value equal to 0 when not. A more comprehensive explanation of this issue is provided in the following section.

The last field of this database contains the length of each polyline in metres. This field was automatically estimated using GIS software and its use is merely analytical.

Continuity across successive editions of Collins’ charts suggest the routes he originally detailed did not change significantly over the period covered by this data. This was a respected source of navigational guidance that was widely used.^12^ Publishers made no revisions to given route information between editions and continued to publish them commercially as dependable guides to those routes [25].^13^ That being so, shifting sandbanks, coastal erosion, and river silting must have affected some coastal route sections.

#### 2.2.1 Lighthouses and visibility ranges

Ships for the most part circumnavigated England and Wales when travelling between ports. They followed routes that, in the main, curved around the country. Navigators depended on the sight of landmarks when coasting, and consequently, these normally had to be visible from sea. This fact allowed us to pinpoint precisely the maximum distance coastal landmarks could be identified from ships everywhere on the coast, which in turn indicates how far away from the shore coasters would normally have sailed. Using GIS, we plotted the maximum distance it was possible to sail from the land before sight of the coastline was lost entirely. We did this by using mathematical principles of earth curvature and sight of horizon from ships. We also introduced night visibility variables based on the location of lighthouses and beacons. This created a realistic outer boundary for the open water routes.

Visibility in daylight and good weather conditions was estimated geometrically based on the Earth's curvature. We assumed an observer located in the mast of a conventional ship at a height h_1_ and a landmark with height h_2_ located on the coast at an elevation z_2_.

Applying the Pythagorean Theorem from the core of the Earth (being R_E_, the radius of the Earth), the horizon line and the two elements described above (observer and landmark), we obtain the following equations:

$$R_{E}^{2}+d_{1}^{2}={\left(R_{E}+h_{1}\right)}^{2}$$

$$R_{E}^{2}+d_{2}^{2}={\left(R_{E}+z_{2}+h_{2}\right)}^{2}$$

Developing these equations, we isolated the visibility from the observer and the landmark:

$$d_{1}=\sqrt{2R_{E}h_{1}+h_{1}^{2}}$$

$$d_{2}=\sqrt{2R_{E}\left(z_{2}+h_{2}\right)+{\left(z_{2}+h_{2}\right)}^{2}}$$

Considering negligible the square of the height of the observer and the square of the elevation and the height of the landmark in comparison to the Earth's radius, we simplify the equations as follows:

$$d_{1}=\sqrt{2R_{E}h_{1}}$$

$$d_{2}=\sqrt{2R_{E}\left(z_{2}+h_{2}\right)}$$

The sum of both equations constitutes the maximum visibility range at which both elements can see each other above the horizon line:

$$range=d_{1}+d_{2}=\sqrt{2R_{E}h_{1}}+\sqrt{2R_{E}\left(z_{2}+h_{2}\right)}$$

We assumed the radius of the Earth as R_E_ = 6,738 km, the average height of an observer aboard a ship as h_1_ = 10m, and the average height of the landmark building as h_2_ = 20 m. We derived the elevation of the terrain on which landmarks were located using [18]. We proceeded to solve the equation for a set of nearly 13,000 locations throughout England and Wales (see Fig. 5).

Note that, assuming the height of the observer on a ship (h1) was equal to zero, the elevation of terrain on the coast (z2) was equal to zero, and the height of the observer (h2) was equal to 2 m, we obtain a visibility range of 4.7 km, i.e. around 3 miles. This limit closely matches the historic three-mile territorial waters limit that was also (probably) determined by the distance ships could be realistically seen from the shore [26]. This historical circumstance supports our visibility method.

To estimate visibility at night-time, the procedure was rather different for the initial and for the final period of study.

For the first period, we proceeded to identify all the lighthouses and beacons recorded by [15].^14^ Each element was then digitised and geolocated using GIS software.

Given the technological limits of lighthouses and beacons at this time, and a lack of consistent information now about those that existed, we opted to digitise all lighthouses and beacons charted by [16]*.*^15^ We assumed that the 16 lighthouses, 10 fire beacons, and 16 unlit beacons we found on Collins' charts amount to a reasonably complete and accurate survey. We found his account to be consistent with authoritative literature on early seamarks and lighthouses [19], [20].^16^ Collins drew sight-lines to some charted lighthouses and beacons, and mariners used these lines to establish their position at sea relative to these structures. We assume the length Collins gave to the lines represented the normal range of visibility for each element. The purported range of the charted sight lines was measured using Collins' own map scales. For lights that Collins attached no sight-line to, we generated average values taken from lighthouses (8.5 km) and beacons (2.5 km) to which he did attach sight-lines. These measures we estimated are close to Naish's range estimates for lights of this kind [20]. Further supporting evidence for light range can be considered, such as the geographical distribution of similar beacons used for inland communication in the county of Kent in the seventeenth century. These beacons were spaced approximately 6.5 km apart [27]. We also discovered that the maximum distance the human eye can spot a candle at night, according to [28], is roughly 3 km, further supporting our estimates for early smaller lights.

To estimate night visibility in the second period we opted for a different strategy. The *Light-Houses of the British Islands*, published in 1832 and 1851 by the UK Hydrographic Office [21], [22], contains highly detailed information about individual lighthouses and light-vessels. Information given includes the appearance of the light (fixed or revolving, and light colour), geographic coordinates, and the number of lights in each single location (some locations had multiple "leading lights"), building height, year of construction, and crucially for us: visibility range in clear weather.

In order to determine the visibility area for lights in the later period, we opted to digitise and geolocate every light type listed and to add a buffer around each to visualise the radius of light visibility detailed by this source. For those elements where authors provided no information, we used the average visibility range of all lights: 21 km.

**Fig. 6:**
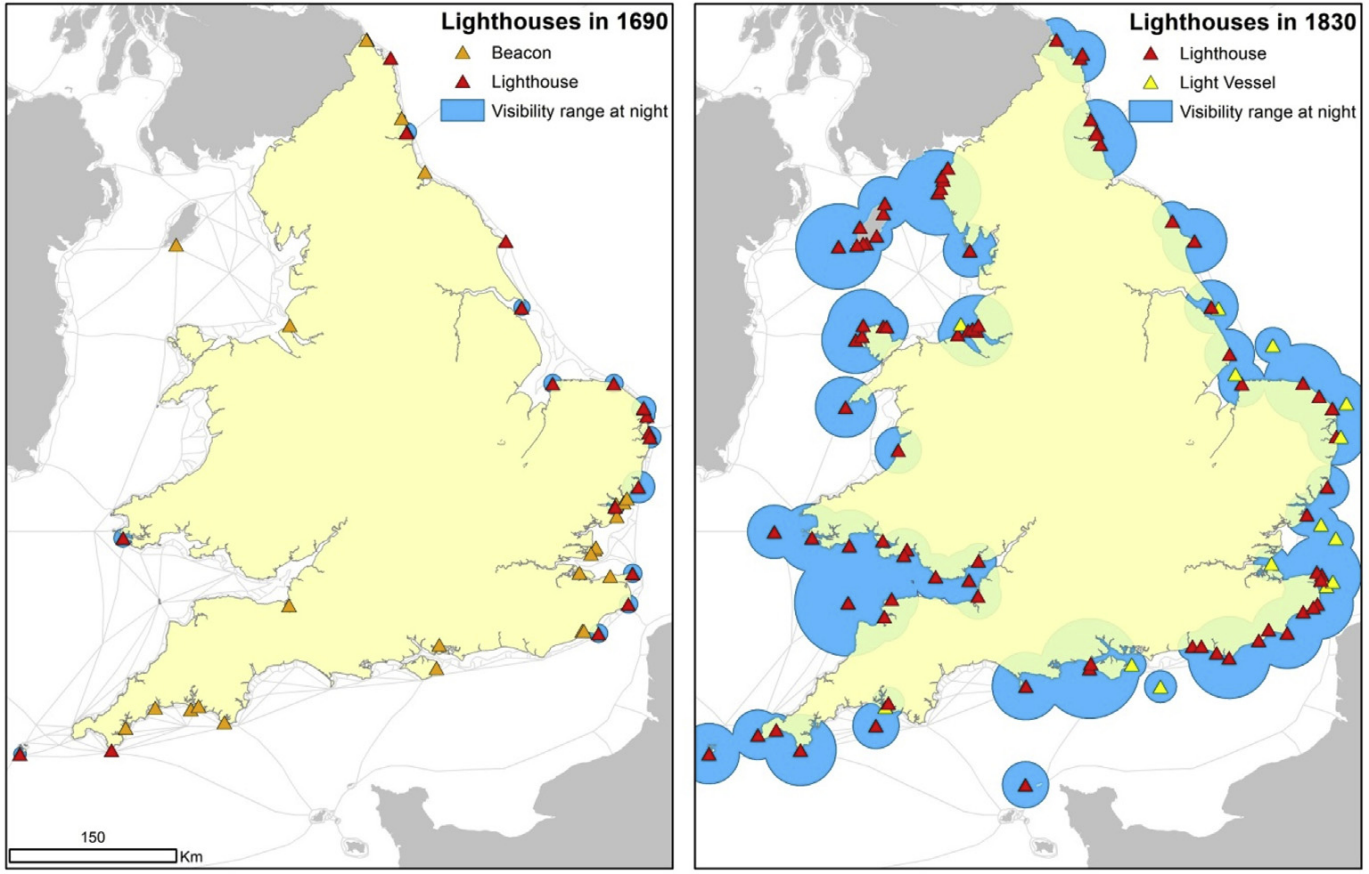
Map of lighthouses with night-time visibility buffers in 1690 (left) and 1830 (right). Own work.

Fig. 6 plots the evolution of lights and visibility range from 1690 to 1830 using these methods. The map visualises the great changes made possible by the extension of lighthouse provisioning, and the technological improvements between these two dates - notably, more powerful lights by the 1830 benchmark. In 1690, the number of lighthouses was relatively small and those that existed were concentrated on the east coast. In 1830, by contrast, all but the remotest coastal areas came to be covered. Lights, where available, aided navigation at night and in foggy conditions, and increased the number of sailing hours available to coasting vessels. Lighthouse development likely reduced shipwrecks, and may have increased shipping speeds.

### 2.3 Technical validation of the network

Once the databases were completed, we proceeded to validate them in GIS using a set of topological rules. The aim of the exercise was to ensure coastal routes connected to points representing ports. In other words, each pair of ports was connected by at least one route. In doing so, we designed the database so other researchers can perform spatial and network analysis by modelling least cost routes through the network.

Database validation required the cleaning of spatially coincident places with different names, as determined by their x and y coordinates. Ports with the same or very close geographical coordinates were removed. Besides which, all landing places had to be connected by a coastal route segment.

The topological rules we used in ArcGIS to do so were the following:

• Must be disjointed
• Must be covered by an endpoint

Regarding coastal routes, we opted for a network composed of individual route segments without intersections or overlap between each. In this case we used the following topological rules in GIS to ensure the validity of the network:

• Must be single-part
• Must not intersect
• Must not overlap
• Must not self-intersect
• Must not self-overlap
• Must not intersect or touch the interior

## Footnotes

1 The authors address England and Wales because these were covered by comparable historical sources. Scotland and Ireland have significantly different records and national histories. With more work, we could however incorporate the ports and routes of these countries.

2 In terms of academic dissemination projects, see ORBIS – The Stanford Geospatial Network Model of the Roman World (http://orbis.stanford.edu/); The Venice Atlas: A Digital Humanities atlas project (http://veniceatlas.epfl.ch/); and Travel in Times: Historic Journey Planner (https://www.travelintimes.org/).

3 We understand landing locations, creeks, harbours, ports, etc. are different, for example in terms of their facilities and scale. However, we do not distinguish them within this paper. Our sources do not provide enough information to deal with their categorisation. For simplicity, we call them all 'ports'. Another ambiguity arises from the term “port” in itself. Some historical sources used the term to refer to customs port, which is the entire stretch of coastline under the jurisdiction of regional customs subsystems. In our case, we linked them to the physical port of the same name.

4 Port books were found in The National Archives, Kew, London, in series E190 and E122. Other interesting sources of shipping movements were identified during the project, such as Lloyd's shipping insurance records, but these often mixed international with coastal shipping, so we ignored these so as not to confuse the very different international and coastal maritime spheres.

5 We assume these recurrent ports may have had historical importance for coastal shipping because of the frequency of their appearance in the sources covering a period between the mid sixteenth century and the early twentieth century.

6 Note that port books did not as a rule mix fishing or international with coastal voyages, meaning the coast port books we used are a record of coasting trade only.

7 Ports were removed from the database if they were located at more than 2 km from a navigable way.

8 In the future, we will replace modern with historical bathymetry data taken from admiralty charts, but in the meantime we consider modern data reliable enough as a long-term indicator of areas with sandbanks and rocks.

9 Beacons were open fires used for navigation and communication.

10 Being: Bristol, Dover, Hull, Liverpool, London, Milford, Penzance, Plymouth, Shoreham, Southampton, Swansea, Weymouth and Great Yarmouth.

11 Specifically, we used 1690 and 1830 as benchmark years.

12 Editions published in 1693 and 1753 were checked at Cambridge University Library.

13 Verner (1969) found few differences between editions.

14 ‘Lighthouses’ are defined here as being single lights and single clusters of leading lights such as at Winterton and Tynemouth. Beacon design as indicated by Collins' depictions varied by location.

15 We use Collins as our main source because it was the first nationally coherent and topographically accurate charting of the coastal waters of the British Isles. Drafted with support of the Admiralty between 1682 and 1693, it includes fine detail for coastal features and infrastructure where these were important to shipping and includes beacons and lighthouses. It is not perfect as a source; detailed description of lights is lacking, and we necessarily relied on simple pictorial evidence Collins gave for these - mainly pictures of small beacons and a few lighthouses. Yet it is the most detailed and reliable source of information for lights for the seventeenth and eighteenth centuries. Another advantage is its national coverage.

16 Except Hunstanton lighthouse, completed in 1665, which was not depicted by Collins.
